# Commentary: Relationship between Milk Microbiota, Bacterial Load, Macronutrients, and Human Cells during Lactation

**DOI:** 10.3389/fmicb.2016.01281

**Published:** 2016-08-17

**Authors:** Tanja Obermajer, Tomislav Pogačić

**Affiliations:** ^1^Department of Animal Science, Biotechnical Faculty, Institute of Dairy Science and Probiotics, University of LjubljanaDomžale, Slovenia; ^2^Dairy Farm–Hanne Storm BremsFaaborg, Denmark

**Keywords:** human colostrum, human milk, microbiota, diversity, microbial interactions, ecosystem homeostasis, functionality

The recent contribution by Boix-Amorós et al. (2016) reveals certain associations among the microbiota, bioactive components and nutrients found in human colostrum and breast milk of 21 healthy Spanish lactating mothers. We would like to emphasize the importance of their findings, discuss some phenomena and highlight the relevant future research directions (Figure 1).

**Figure 1:**
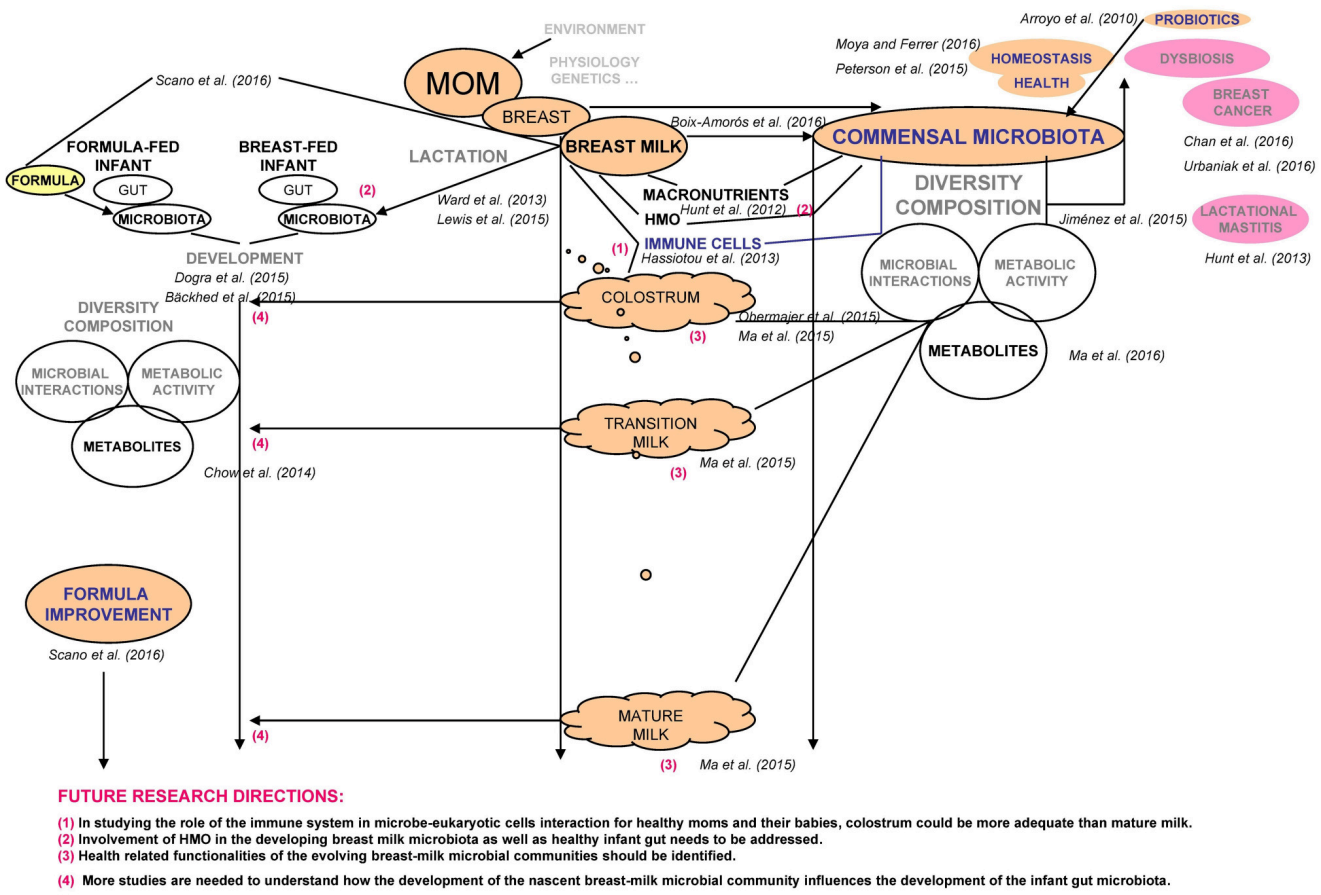
**Schematic presentation summarizing the discussed current research work and proposed future research directions**.

The commensal microbiota, as part of epigenetic landscape, reflects human health status by its diversity, composition and metabolic activity (Moya and Ferrer, 2016). An improved understanding of the factors controlling health associated bacterial interactions may, upon perturbation, define novel therapeutic or preventive measures to restore individual's dynamic microbial equilibria and immune homeostasis (Peterson et al., 2015). Probiotics were demonstrated as an effective tool in lactational mastitis interventions (Arroyo et al., 2010), and may even hold potential in breast cancer prevention (Chan et al., 2016; Urbaniak et al., 2016).

In the study by Boix-Amorós et al. (2016), microbial communities, again, were confirmed as complex and individual-specific. High inter-subject variability was disclosed for the composition and the number of bacteria per ml, while higher total bacterial load was not associated with lower diversity of milk sample. Namely, as reported previously, the milk microbiome of mastitis-affected women suffered a loss of bacterial diversity, while an increase in sequences related to the presumptive etiological agents was also observed (Jiménez et al., 2015). Staphylococci and streptococci were classified in dominant core of genera, existing at all three lactation sampling points (Boix-Amorós et al., 2016). The study further suggested that the specific microbiota composition, rather than total sample bacterial load, might trigger the mammary gland immune responses. Moreover, the positive correlation was only established between the staphylococci proportion and somatic cell count (SCC). Specifically, the SCC biomarker was earlier reported (Hunt et al., 2013) to be roughly 10-fold increased in milk from mastitic compared to milk from healthy breasts. Similarly to the work by Boix-Amorós et al. (2016), our recent publication studied the colostrum bioactive potential of 45 healthy Slovenian mothers (Obermajer et al., 2015) and qPCR approach was applied on the automatedly extracted colostrum microbial DNA. The interactions among target bacterial groups were investigated. As in previous reports concerning microbial biofilms, but in oral specimens (Benítez-Páez et al., 2014; Loozen et al., 2014), here, we would like to underline the positive abundance correlations found between the specific microbial components in colostrum samples of different mothers (e.g., *Staphylococcus* and total bacterial load; *Staphylococcus* and *Staphylococcus epidermidis*), testifying that some common issues in microbial consortia truly exist and may play a role in the maintenance of the ecosystem homeostasis (Obermajer et al., 2015). As suggested by Delgado et al. (2008), the trend toward the overgrowth of one component (e.g., staphylococci and streptococci strains as a common mastitis causing agents) could provoke a dysbiotic process in a predisposed host. Interestingly enough, staphylococci and streptococci were also quantitatively correlated (Spearman's rank correlation coefficient 0.478; *p* < 0.01) (Obermajer et al., 2015), even though their quantities varied considerably among samples of different mothers. In contrast, Boix-Amorós et al. (2016) reported a trend toward the negative association between the total bacterial load and the staphylococci proportion. While the difference in this correlation remains to be elucidated, we could hypothesize that a significantly higher level of immune cells in colostrum than in mature milk (up to 70% of the total milk cells for colostrum vs. 0–2% of total cells for mature milk; Hassiotou et al., 2013) could favor different associations between specific microbial populations in both human fluids. Thus, differential characteristics of colostrum, mainly related to immune cells, could make this biological samples more suitable to study bacteria–human immune cells interactions.

The study by Boix-Amorós et al. (2016) presented different compositional and diversity patterns in milk microbiome at three lactation stages. Considering few bacterial genera present at all-time points, future research should focus on the bacterial species interaction networks as a research tool to decipher possible functionalities changing during evolution of milk microbial communities. The robust microbiotas, able to regain their original function upon disturbances, should be identified. The proposed networking approach on the breast milk dataset was recently employed for the geographically distinct healthy cohort (Ma et al., 2015) and was also applied (Ma et al., 2016) in the preliminary comparative analysis of Hodgkin's lymphoma milk microbiome after drug administration. Moreover, the authors suggested the metabolites-microbial species networks to be very useful in identifying the active bacterial taxa really involved in the milk microbial communities conferred to infants.

Boix-Amorós et al. (2016) successfully identified some associations among macronutrients and specific microbial genera, however, the authors failed to address an important prebiotic component: human milk oligosaccharides (HMO). Hunt et al. (2012) demonstrated that HMO stimulated the growth of staphylococci isolates from breast milk. But, the observed effect was only associated with the increased amino acid metabolism and not with the depletion of HMO. As proposed, the specific HMO profiles in women may be involved in the development of lactational mastitis. Lewis et al. (2015) extended the importance of the maternal synthesis of human milk glycans on the bifidobacterial species content in the evolving infant gut.

Daily consumption of 10^7^–10^8^ bacterial cells by a breast-fed infant (Boix-Amorós et al., 2016) is a valuable information from the perspective of competitive early supplementation of infant formulae. Ward et al. (2013) suggested that a broad array of microbes within the human milk likely contributes toward an effective infant gut colonization, while diverse microbial DNA sequences as well as mother's DNA may create a balanced immune stimulatory and suppressive DNA exposures, leading infants to eventual tolerance of the immense number of gut bacteria. Following birth, the microbial gut consortia form in a non-random, successive fashion (Dogra et al., 2015). While the developing microbiome of formula-fed infants fails to resemble the compositional and functional capacity of the microbiome of breast-fed infants (Bäckhed et al., 2015), the proposed research directions (Figure 1) with network analyses (nutrients-microbes-metabolites; Chow et al., 2014; Scano et al., 2016) may open new frontiers to formulate the next generation of innovative infant foods.

## Author contributions

TO drafted the work and TP critically revised the work. Both authors approved the final version.

### Conflict of interest statement

The authors declare that the research was conducted in the absence of any commercial or financial relationships that could be construed as a potential conflict of interest.
